# Plant Flavonoids: Chemical Characteristics and Biological Activity

**DOI:** 10.3390/molecules26175377

**Published:** 2021-09-04

**Authors:** Maria Celeste Dias, Diana C. G. A. Pinto, Artur M. S. Silva

**Affiliations:** 1 Centre for Functional Ecology, Department of Life Sciences, University of Coimbra, Calçada Martim de Freitas, 3000-456 Coimbra, Portugal; 2 LAQV/REQUIMTE, Department of Chemistry, Campus Universitário de Santiago, University of Aveiro, 3810-193 Aveiro, Portugal; diana@ua.pt (D.C.G.A.P.); artur.silva@ua.pt (A.M.S.S.)

**Keywords:** antioxidants, bioactive compounds, extraction methods, natural sources, structure

## Abstract

In recent years, more attention has been paid to natural sources of antioxidants. Flavonoids are natural substances synthesized in several parts of plants that exhibit a high antioxidant capacity. They are a large family, presenting several classes based on their basic structure. Flavonoids have the ability to control the accumulation of reactive oxygen species (ROS) via scavenger ROS when they are formed. Therefore, these antioxidant compounds have an important role in plant stress tolerance and a high relevance in human health, mainly due to their anti-inflammatory and antimicrobial properties. In addition, flavonoids have several applications in the food industry as preservatives, pigments, and antioxidants, as well as in other industries such as cosmetics and pharmaceuticals. However, flavonoids application for industrial purposes implies extraction processes with high purity and quality. Several methodologies have been developed aimed at increasing flavonoid extraction yield and being environmentally friendly. This review presents the most abundant natural flavonoids, their structure and chemical characteristics, extraction methods, and biological activity.

## 1. Introduction

Flavonoids are secondary metabolites that are very abundant in plants, fruits, and seeds, responsible for the color, fragrance, and flavor characteristics. In plants, flavonoids perform many functions like regulating cell growth, attracting pollinators insects, and protecting against biotic and abiotic stresses [1]. For instance, plant flavonoids can operate as signal molecules, UV filters, and reactive oxygen species (ROS) scavengers and have several functional roles in drought, heat, and freezing tolerance [2,3,4]. In humans, these compounds are associated with a large range of health benefits arising from their bioactive properties, such as anti-inflammatory, anticancer, anti-aging, cardio-protective, neuroprotective, immunomodulatory, antidiabetic, antibacterial, antiparasitic, and antiviral properties [5,6,7]. However, flavonoids’ chemical structure, particularly hydroxy groups’ presence, influences humans’ bioavailability and biological activity [8]. Flavonoids possess a basic 15-carbon flavone skeleton, C6-C3-C6, with two benzene rings (A and B) linked by a three-carbon pyran ring (C). The position of the catechol B-ring on the pyran C-ring and the number and position of hydroxy groups on the catechol group of the B-ring influence the flavonoids’ antioxidant capacity [9]. The functional hydroxy groups in flavonoids can donate electrons through resonance to stabilize free radicals and mediate antioxidant protection [10]. Based on the structure of the flavonoids, they can be classified into six major classes, flavan-3-ols, flavones, flavonols, flavanones, isoflavones, and anthocyanins [10]. Due to their remarkable antioxidant characteristics, flavonoids are used in the food, cosmetic, and pharmaceutical industries [11]. However, the industrial use of these antioxidants implies extraction processes with high purity and quality. Therefore, several procedures for the extraction of flavonoids have been explored, and in recent years more environmentally friendly extraction methods and strategies that achieve high yields have been developed [1].

## 2. Natural Occurrence of Flavonoids

### 2.1. Flavonoids Biosynthesis, Structure, and Classification

**Figure 1:**
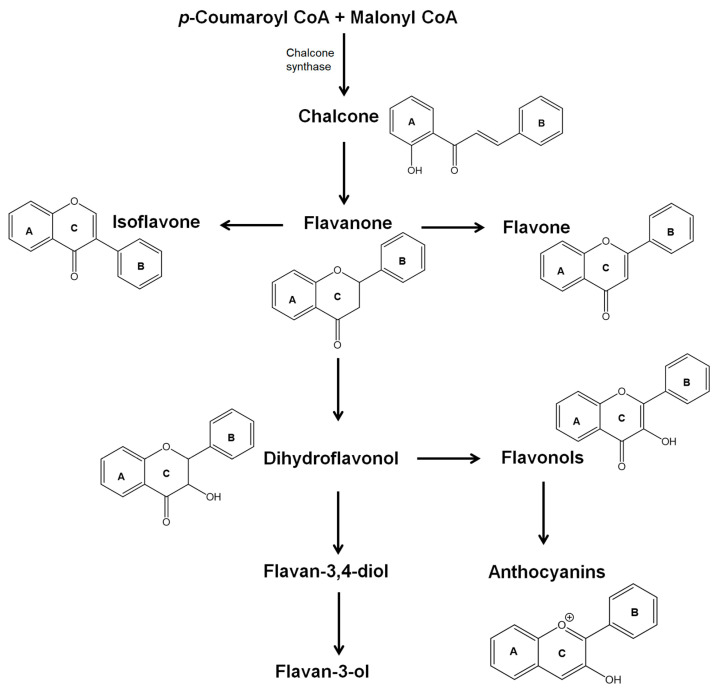
Flavonoid biosynthesis.

Flavonoids are included in the large family of phenolic compounds or polyphenols and comprise more than 6000 different structures [10]. In plants, flavonoids are derived from two biosynthetic pathways, the phenylpropanoid, which produces the phenylpropanoid skeleton (C6-C3), and the polyketide that produces blocks for polymeric C2 units [12]. The enzyme chalcone synthase catalyzes the formation of the 2′-hydroxychalcone scaffold, scientific name (*E*)-1-(2-hydroxyphenyl)-3-phenylprop-2-en-1-one (Figure 1), from *p*-coumaroyl CoA and malonyl CoA, which are then used in several enzymatic steps to produce other flavonoids [13]. Several factors, such as environmental conditions (e.g., light, water availability, and temperature), hormones (e.g., jasmonic acid), and physical injuries, influence the expression of the genes involved in flavonoid biosynthesis leading to changes in their availability [14].

Almost all flavonoids present a C_6_-C_3_-C_6_ structure containing two benzene rings, A and B, connected by a heterocycle pyrene ring (C) that contains oxygen. Flavonoids can be divided into two major categories depending on the degree of central heterocyclic ring saturation [5]. For example, anthocyanidins, flavones, flavonols, and isoflavones present a C2=C3 unsaturation, whereas flavanones, dihydroflavonols, and flavan-3-ols are examples of saturated flavonoids (Figure 1). Although this classification is the most common one, flavonoids can also be classified based on molecular size, primarily due to the prevalence of biflavonyls in gymnosperms. Another important point regarding flavonoid structure is the degree of substituents on the A and B rings, such as hydroxy, alkyl, and methoxy.

Furthermore, in plants, flavonoids may be found in the free form (aglycones) or linked to sugars. Actually, these glycosylated flavonoids are most common, and, for example, the glycosylated anthocyanidins are recognized as an essential class of flavonoids, anthocyanins. In fact, anthocyanidins are light-sensitive and are found linked to sugars. The most abundant form of flavonoid glycosides is *O*-glycosides, but *C*-glycosides can also be found [10]. Glycosylation enhances solubility, distribution, and metabolism by facilitating transport through the membrane, and methylation increases the entry of flavonoids into the cells and protect them [10].

#### 2.1.1. Anthocyanins

**Figure 2:**
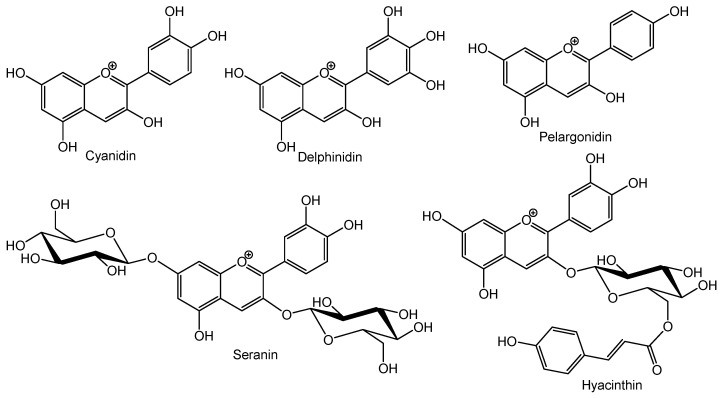
Examples of representative anthocyanidins and anthocyanins.

Anthocyanins are responsible for a flower’s colors, from pink to blue, but they are also present in leaves, fruits, and roots. They are prevalent in angiosperms, although their occurrence in gymnosperms has also been reported [15]. Anthocyanins are also recognized for their biological properties, which, together with their bright color, make them interesting additives for food preparations [16,17]. From a chemical point of view, and as mentioned above, anthocyanins are the anthocyanidins *O*-glycosides. Anthocyanidins (Figure 1), highly oxidized 2-aryl-3-hydroxychromenylium, are also colored pigments, although less stable, and consequently few examples are found in nature, the most widespread derivatives being cyanidin, responsible for red to magenta colors, delphinidin, responsible for purple to blue colors, and pelargonidin, responsible for orange to pink colors (Figure 2). This color differentiation may be associated with one of the main rules of these flavonoids in plants, which is to attract animals for pollination, and different colors attract different animals. In addition, the presence of a sugar moiety promotes some changes in color brightness. The most common sugar is glucose with a β-linkage, but galactose, rhamnose, and xylose are also found. Moreover, these sugar moieties can have acyl substituents, highlighting cinnamic acyl derivatives, such as caffeic, ferulic, and *p*-coumaric acids, due to these phenolic acid rules in plants’ antioxidant activity.

#### 2.1.2. Flavanones and Dihydroflavonols

**Figure 3:**
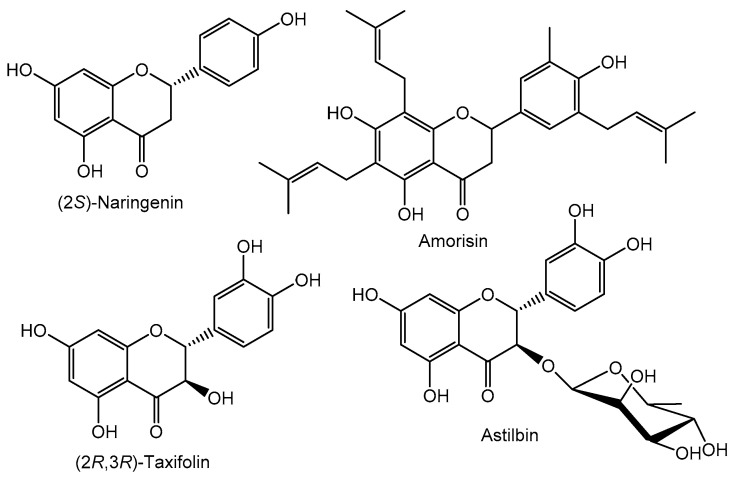
Examples of representative flavanones and dihydroflavonols.

Flavanones, 2-arylchroman-4-ones (Figure 1), are obtained through the isomerization of 2′-hydroxychalcones by a ring closure, which produces a stereogenic center at carbon C-2. Therefore, naturally occurring flavanones are optically active and mainly with a (2*S*) stereogenic configuration as in the case of naringenin (Figure 3), a common scaffold among the natural flavanones [15]. Many natural flavanones are also linked to sugars, usually in the form of 7-*O*-glycosides, but several examples present prenyl side chains [18].

Dihydroflavonol, 2-aryl-3-hydroxychroman-4-one (Figure 1), biosynthesis involves an oxidative hydroxy group addition at flavanones’ C-3 position, which is the reason why they are often designated as 3-hydroxyflavanones. One of the most common derivatives that is also the primary scaffold for several other naturally occurring dihydroflavonols is taxifolin (Figure 3). These flavonoids are also found linked to sugars, an important example being astilbin, which shows remarkable anti-inflammatory activity [19] and is linked to other groups, such as prenyl and methoxy groups.

#### 2.1.3. Isoflavones

**Figure 4:**
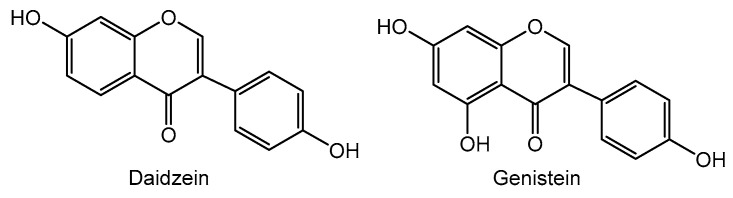
Examples of representative isoflavones.

Isoflavones, 3-aryl-4*H*-chromen-4-ones (Figure 1), are obtained from flavanones through a rearrangement that promotes 2,3-aryl migration, followed by a dehydrogenation. Although it is commonly said in the literature that the designation isoflavonoids results from the isolation of other compounds, such as isoflavanones or isoflavans, isoflavones remain the most common in nature. Isoflavones’ occurrence is still restricted to a few subfamilies of the Leguminosae family [20]; nevertheless, important estrogenic activity is attributed to these metabolites [21], and some medicinal plants’ anti-inflammatory properties are a result of their richness in isoflavones [22]. The most common scaffold is daidzein and genistein (Figure 4), which are also found linked to sugars, although there are only a few examples [15].

#### 2.1.4. Flavones and Flavonols

**Figure 5:**
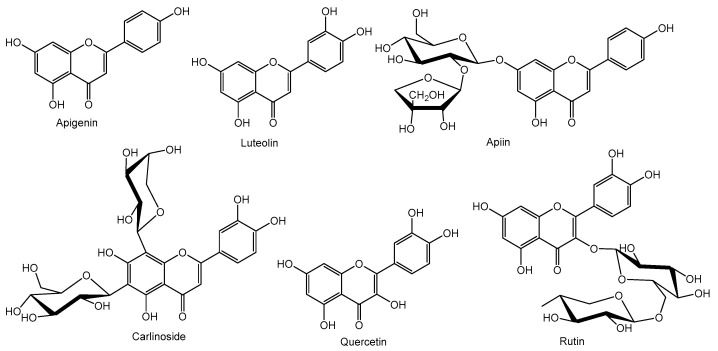
Examples of representative flavones and flavonols.

Flavones, 2-aryl-4*H*-chromen-4-ones, and flavonols, 2-aryl-3-hydroxy-4*H*-chromen-4-ones (Figure 1), are obtained through dehydrogenation of flavanones and dihydroflavonols, respectively. Flavones are widespread and are the most representative class of flavonoids, moreover if it is considered that flavonols are 3-hydroxyflavones. Due to their prevalence in nature, along with their recognized biological activities, flavones have gathered the interest of scientists [23]. According to their substitution pattern and their large distribution, flavones are further subdivided into classes, such as *O*-methylated, *C*-methylated, and isoprenylated, among others [15]. Among the flavonoid family, flavones are the ones that occur both as *O*- and *C*-glycosides, being the most spread aglycones the apigenin and the luteolin (Figure 5).

The first isolated *O*-glycoside was apiin, an apigenin 7-apiosylglucoside (Figure 5), and since its isolation, several derivatives have been uncovered. Although examples with other sugar moieties are found, glucose is the most common, and the flavone preferable *O*-glycosylation site is C-7. Interesting is the fact that more than one sugar unit can be attached, even in cases of *C*-glycosides, as can be seen in carlinoside, a luteolin 6-glucoside-8-arabinoside (Figure 5).

The most prevalent flavonol is quercetin (Figure 5), for which several biological properties have been established [24], and which occurs both in the aglycone and *O*-glycoside forms. *O*-glycosylation occurs at C-3, which is the case of rutin (Figure 5), probably the most widespread flavonol glycoside in the plant kingdom [15].

### 2.2. Sources of Flavonoids

Flavonoids can be found in several beverages and foods, like wine, beer, and tea, but fruits, vegetables, flowers, and seeds are the sources with the highest amounts of natural flavonoids [25]. However, the amount of these compounds depends on several factors, such as plant cultivar/genotype, growing environment conditions, soil characteristics, harvest, and storage.

**Figure 6:**
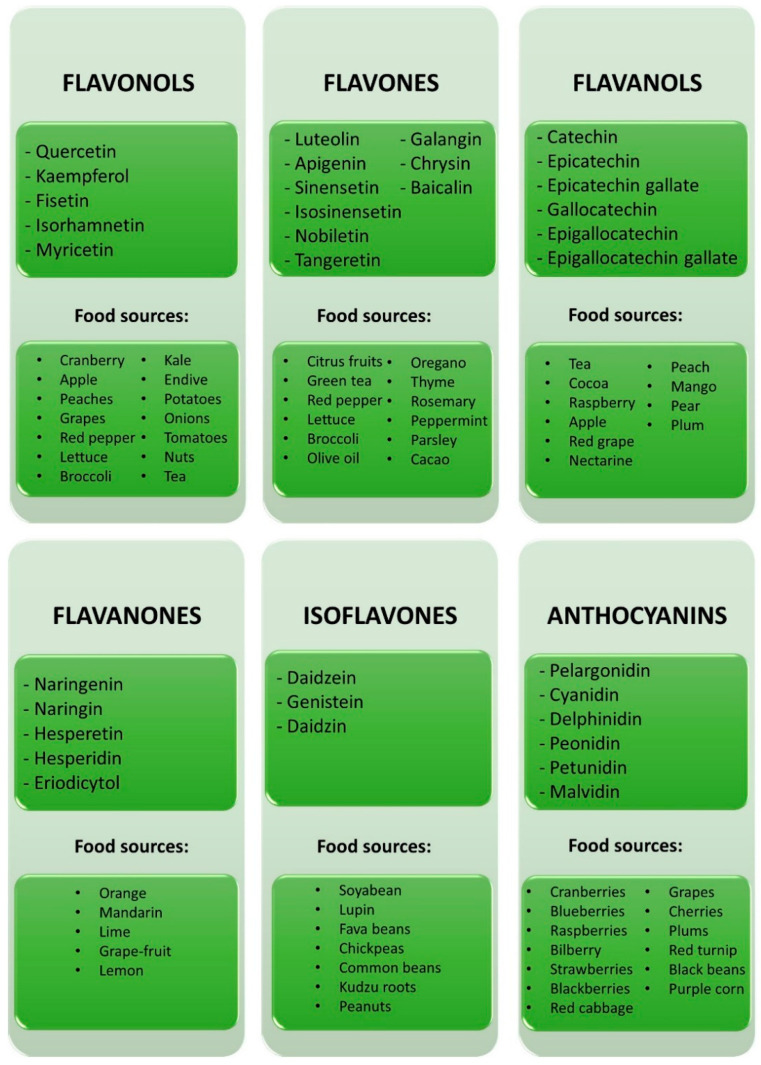
Flavonoid classes and examples of natural food sources.

Flavonols that comprise, for example, quercetin, kaempferol, fisetin, isorhamnetin, and myricetin are abundant in green leaves, fruits, and grains [26,27] (Figure 6). For instance, lettuce, cranberry, apple, peaches, and red pepper are rich in quercetin and kaempferol [3,28]. Spinach leaves have high amounts of rutin, spinacetin glycosides, and patuletin glycosides, while broccoli, kale, endive, potatoes, onions, grapes, and tomatoes contain more kaempferol 3-*O*-glycosides [3]. Myricetin can be found in nuts, berries, tea, and also red wine [3,29].

Flavones are among the most important flavonoids and are represented by luteolin, apigenin, sinensetin, isosinensetin, nobiletin, tangeretin, galangin, and chrysin [26] (Figure 6). These compounds can be mainly found in leaves, flowers, and fruits as glucosides of apigenin, luteolin, and diosmetin [27]. For instance, celery is rich in apigenin 7-*O*-glycoside, and the glycosides of luteolin and apigenin are abundant in several citrus fruits, green and red peppers, lettuce, broccoli, olive oil, cacao, oregano, thyme, rosemary, peppermint, and parsley [3].

Flavanols, or flavan-3-ols, comprise catechin, epicatechin, epicatechin gallate, gallocatechin, epigallocatechin, and epigallocatechin gallate [30] (Figure 6). Flavanols are found in high concentration in *Camellia sinensis*, tea plant, as (-)-epigallocatechin gallate, (-)-epicatechin gallate, (-)-epigallocatechin, and (-)-epicatechin, tea consumption being one of the most important sources of these flavonoids [31]. In addition, fruits like apples, red grapes, peaches, mangoes, pears, plums, nectarines, and raspberries are very rich in (+)-catechin, (-)-epicatechin, and (-)-epigallocatechin. Cocoa and red wine are good sources of catechins [3,28].

Flavanones, also known as dihydroflavones, are an important class of flavonoids widely found in citrus fruits (Figure 6). For instance, flavanone glycosides, like naringin, naringenin, and naringenin 7-*O*-neohesperidoside, can be found in grapefruits, hesperidin, hesperetin, and hesperetin 7-*O*-rutinoside in oranges, mandarins, limes, and lemons, and eriocitrin, eriodictyol, and eriodictyol 7-*O*-rutinoside in lemons [27,28].

Isoflavones have a stricter distribution in plants, being predominantly produced in legumes [32]. Genistin, glycitin and daidzin glycosides, and malonylated isoflavones are particularly found in soybeans [3] (Figure 6). Lupin, fava beans, and kudzu roots also contain genistin. Small quantities of isoflavones are present in common beans, peanuts, and chickpeas [32].

Anthocyanins are the flavonoids responsible for the blue, purple, red, and orange color of several flowers, leaves, and fruits. This class of compounds is commonly present as glycosides of anthocyanidins, such as cyanidin, pelargonidin, delphinidin, peonidin, petunidin, and malvidin [17,28] (Figure 6). For example, cranberries, blueberries, raspberries, bilberries, strawberries, blackberries, plums, grapes, cherries, and sweet potatoes have high amounts of anthocyanins [3]. Vegetables such as red cabbages, red turnips, and purple sweet potatoes are rich in acylated anthocyanins. In addition, black beans and purple corn have cyanidin 3-*O*-glucoside [27]. The blue color of some flowers is due to delphinidin, while orange color is associated with pelargonidin [17].

Natural flavonoids can be extracted and used in the food industry instead of synthetic compounds to improve food quality. In recent years, the restriction imposed on the use of some synthetic antioxidants, such as the case of butylated hydroxyanisole (BHA), butylated hydroxytoluene (BHT), and propyl gallate, increased the interest in natural flavonoids mostly due to their capacity to retard oxidative degradation of lipids, improve the quality and nutritional value of food, and reduce toxicity [33]. Flavonoids can be used as food preservatives, preventing lipid oxidation and protecting vitamins and enzymes, inhibitors of microbial growth in foodstuffs, additives in human dietary supplements and animal feeds, flavorings, and colorants (e.g., anthocyanins) [33]. Some flavonoids also inhibit fungal spore germination and have been proposed to be used as a fungal pathogen control agent in some foodstuffs [34]. Flavonoids are very versatile, displaying photochemical properties that can be used to protect beverages against light-induced color deterioration [35]. Since flavonoids are natural compounds with low toxicity, very abundant in plants, and inexpensive, their increased use as food additives in place of synthetic preservatives will contribute to the food industry’s sustainability.

## 3. Extraction, Isolation, and Characterization of Flavonoids

The use of antioxidants from natural sources in the food, cosmetic, and pharmaceutical industries involves comprehensive know-how of the extraction processes suitable for obtaining high purity and quality extracts. Therefore, it is crucial to design new extraction methodologies or improve old ones.

The solubility of flavonoids in different solvents varies, so the solvents are chosen according to flavonoids’ polarity. For instance, aglycones highly alkylated are preferably extracted with ethyl acetate. It should be emphasized that toxic solvents, such as benzene and chloroform, early used [36], are not recommended and may even be forbidden. On the other hand, more polar aglycones such as hydroxylated ones and glycosides are preferably extracted with acetone, alcohol, water, or mixtures of these solvents [36,37].

Besides the solvents, the methodologies are also critical. The conventional methods, although less green, are still used chiefly because they employ low-cost equipment. From these, frequently used are maceration and Soxhlet extraction as they do allow the isolation of flavonoids, but they also present several disadvantages, including the use of a large quantities of solvents, long extraction times, and high energy consumption [1,38]. Furthermore, these conventional techniques require several subsequent purification steps to obtain the pure compound, so more and more scientists are developing new approaches to extract more efficiently the secondary metabolites. The so-called unconventional techniques or green extraction methods include mainly ultrasound-assisted extraction (UAE), microwave-assisted extraction (MAE), supercritical fluid extraction (SFE), and pressurized liquid extraction (PLE) [1,38,39,40,41], but a few other techniques have also been developed and are being used, such as enzyme-assisted extraction (EAE), matrix solid-phase dispersion (MSPD) [38], pulsed electric field (PEF) [40], and solid-state fermentation (SSF) [42], among others [38,40].

The unconventional extraction techniques present more advantages (e.g., lower consumption of organic solvents and extraction time) when compared to conventional techniques and are very effective in the extraction of flavonoids from several types of natural matrices [43,44]. The UAE technique is based on the application of ultrasounds in the kilohertz range (20–100) that move through the solvent, causing cavitation bubbles. When the cavitation bubbles burst at the surface of the samples, damage to the cell wall leads to cell disruption or disintegration. It, therefore, enhances the penetration of solvents into the cells, improving the release of compounds [39,40,41]. In the MAE technique, the application of nonionizing electromagnetic waves with frequencies between 300 MHz to 300 GHz induces disruption or changes in the structure of sample cells [41,44]. The extraction solvent must be polar to absorb microwave energy. This extraction method involves increasing temperature and pressure, resulting in solute separation from the sample, followed by solute released to the solvent and solvent diffusion through the sample [39,40,41].

Besides the above-mentioned methods, SFE attracts several scientists because it uses a fluid above its critical pressure and temperature, increasing its solvation power and, consequently, the compounds’ solubility. Moreover, separating the extract from the solvent is usually straightforward, involving decreasing the pressure and release of the solvent to the atmosphere. At the same time, the extract is collected in a vessel. Supercritical carbon dioxide (scCO_2_) is the fluid mainly used to extract phenolic compounds. It has a mild critical pressure (74 bars) and low critical temperature (31 °C), and is considered safe because it is nonflammable, noncorrosive, environmentally friendly, and inexpensive. scCO_2_ has been used in the extraction of phenolic compounds [45], although its combination with ethanol increases the extraction of flavonoids [46].

Finally, we can highlight PLE, a process that employs high pressure to maintain the solvent in the liquid state at temperatures above its boiling point but below the critical point, consequently increasing the compounds’ extraction [47]. Literature analysis indicates that flavonoids can be extracted using mixtures of water and ethanol at temperatures between 40 to 80 °C and pressures around 100 bar [48].

Although this review does not provide a detailed analysis of these methodologies, it seems evident that they have been applied with some success to extract flavonoids; usually, a mixture of flavonoids is obtained. Some experiments suggest that UAE and MAE do not improve flavonoid extraction [49], leading us believe that the most crucial aspect is the solvent used in the extraction. In this regard, it seems there is consensus that deep eutectic solvents (DES) are highly recommended to improve flavonoid extraction [50]. Moreover, these solvents can be employed to target specific flavonoids [51].

**Figure 7:**
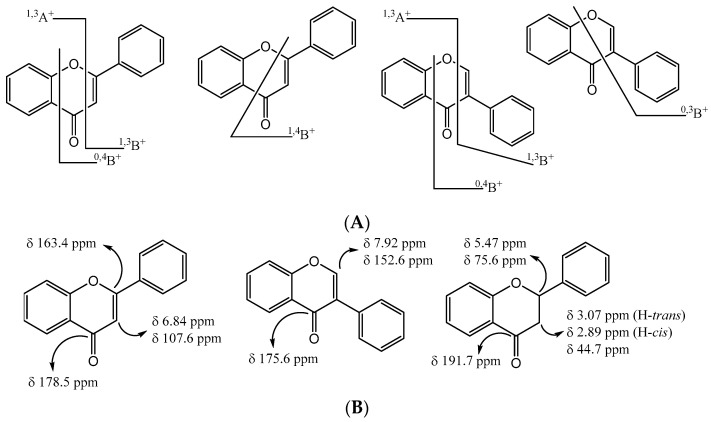
(**A**): The most important ion fragments of flavones and isoflavones. (**B**): The most important NMR signal nucleus.

Nowadays, flavonoid characterization is mainly achieved using nuclear magnetic resonance (NMR) and mass spectrometry (MS), with the significance of MS enlarged with the development of liquid chromatography techniques coupled with MS. However, some crucial structural characterization can also be achieved by infrared spectroscopy and ultra-violet (UV) absorption spectroscopy data [43], which is also important in liquid chromatography analysis. Concerning the importance of the MS fragmentation analysis, several publications detailed flavonoid fragmentation [52,53] from which some main fragments can be highlighted (Figure 7A). Most of them involved cleavage of the heterocyclic ring, which allowed the identification of the substituents in the flavonoid nucleus. The typical fragmentation joined with UV data is the basis of several studies involving the establishment of the flavonoid profile of several extracts. Important information concerning flavones and flavonols was recently published [54]. Furthermore, these data types are also being gathered on the less studied isoflavones [55,56].

Flavonoid structure elucidation using NMR is most common and allows the identification of the different scaffolds mainly due to the heterocyclic ring protons and carbons’ typical chemical shifts [57]. For example, isoflavones can be differentiated by H-2 and C-2 chemical shifts, which differ from those of H-3 and C-3 chemical shifts of flavones (Figure 7B). Furthermore, the flavanone nucleus without the C2=C3 carbon–carbon double bond also presents typical chemical shifts that allow their identification (Figure 7B) [43].

## 4. Metabolism and Biological Activity of Flavonoids

### 4.1. Metabolism and Bioavailability

The knowledge of the content of flavonoids in foods is not enough to provide the information of their bioefficacy in human health [7,58]. This is because flavonoid bioavailability is low due to restrictions in absorption, modifications throughout the gastrointestinal tract caused by microorganisms, chemical and mechanical effects, and rapid excretion [58].

Most of flavonoids, except flavanols, present as glycosides in food, and glycosylation influences absorption. Only flavonoid aglycones and some glucosides can be easily absorbed in the small intestine [58]. Most of flavonoid glucosides must be deglycosylated in the small intestine before absorption, but the rate of deglycosylation depends on the structure and position of the sugar substitution [59]. The flavonoids not deglycosylated are hydrolyzed by enzymes in the colon and reabsorbed or eliminated in feces [60,61]. The flavonoids absorbed are conjugated in the liver cells by glucuronidation, sulfation, and methylation [60]. From the liver, some metabolites are distributed in the blood (delivered to target tissues and organs where they exert their bioactivity). In contrast, others are secreted into the bile and undergo enterohepatic recirculation, or they can be eliminated by urine [60,62].

The bioavailability of flavonoids depends on their class but is, in general, very low. For instance, isoflavones are reported as the most bioavailable flavonoid, more absorbed in the intestine, while other flavonoids, such as galloylated catechins and anthocyanins, are poorly absorbed [7,59,62].

### 4.2. Biological Activities

Scientific evidence has shown that flavonoids induce several health benefits in humans, and a diet rich in these compounds can help prevent some chronic diseases [6,7,25].

Flavonoids present several properties, but the one related to the ability to scavenge free radicals and act as antioxidants is indubitably the most relevant. Within flavonoids classes, the antioxidant capacity varies depending on the type of functional group and its arrangement around the nuclear structure [61]. The number and position of the hydroxy groups in the catechol B-ring and their position on the pyran C-ring influence the free radical scavenging ability [9]. The functional hydroxy group of the structure can donate an electron and hydrogen to a radical through resonance, stabilize them, and originate a relatively stable flavonoid radical [4].

The antioxidant action mechanisms of flavonoids can be by the (a) direct scavenging of ROS, (b) inhibition of ROS formation through the chelation of trace elements (e.g., quercetin has iron-chelating and iron-stabilizing properties), or inhibition of the enzymes that participate in the generation of free radicals (e.g., glutathione *S*-transferase, microsomal monooxygenase, mitochondrial succinoxidase, NADH oxidase, and xanthine oxidase), and (c) activation of antioxidant defenses (e.g., upregulation of antioxidant enzymes with radical scavenging ability) [11,28,61]. A combination of some of these mechanisms, for example, radical scavenging action with suppression of some enzyme functions, may also occur [11]. Most of the flavonoids appear as glycosides, and the number and position of connections with the sugar affect the antioxidant properties of the flavonoid [60]. However, aglycone forms have a higher antioxidant capacity, but their availability is lower.

**Figure 8:**
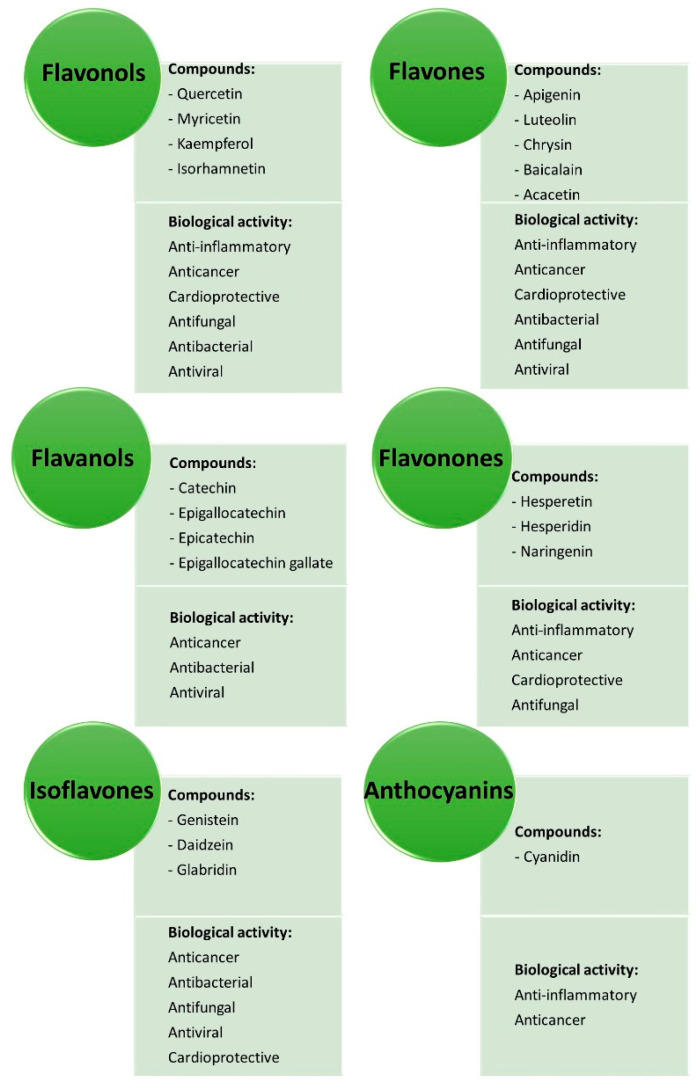
Flavonoid classes and some examples of their biological activities.

In addition to antioxidant properties, many other actions of flavonoids are described, such as anti-inflammatory, anticancer, cardioprotective, antimicrobial, and antiviral (Figure 8).

#### 4.2.1. Anti-Inflammatory Action

Inflammation occurs in response to several causes, such as a tissue physical injury or trauma, chemical exposure, and microbial infection. Usually, the inflammation process is rapid and self-limiting, but in some cases, prolonged inflammation periods contribute to the development of several chronic or degenerative disorders like cancer, diabetes, cardiovascular and neurodegenerative diseases, and obesity [8]. In an inflammatory process, flavonoids can act as: (a) antioxidants scavenging ROS or reducing free radical accumulation, (b) inhibitors of the activity of regulatory enzymes (e.g., protein kinases and phosphodiesterase) and transcription factors related to the control of mediators involved in the inflammatory process, and (c) modulators of the activity of the immune cells (e.g., inhibition of cell activation, maturation, signaling transduction, and secretory processes) [8,11].

Both genetic and environmental factors exert an important role in the inflammation process. Several studies have demonstrated that an active lifestyle together with a healthy diet, rich in fruits and vegetables as well as non-processed and low-sugar foods, prevent inflammatory diseases [8]. Some flavonoids, such as flavonols (e.g., quercetin, rutin, and morin), flavanones (e.g., hesperetin and hesperidin), flavanols (e.g., catechin), isoflavones (e.g., genisten), and anthocyanins (e.g., cyanidin) have been demonstrated to exhibit anti-inflammatory functions during in vitro and in vivo experiments and in clinical studies [6,63].

#### 4.2.2. Anticancer Action

The anti-inflammatory properties of flavonoids also have an important impact on cancer development. These compounds exert their activity by inactivating carcinogen, inducing apoptosis, triggering cell cycle arrest, and inhibiting angiogenesis [27]. Flavonoids have been reported to inhibit tumor cell proliferation by inhibition of ROS formation and repression of the enzymes xanthine oxidase, cyclooxygenase-2, and 5-lipoxygenase, implicated in tumor promotion and development [63].

Flavonoids exert a wide range of anticancer effects. For instance, the flavonoids isorhamnetin and acacetin can inhibit the proliferation of human breast cancer [8]. Kaempferol possesses antiproliferative and apoptosis activity in human osteosarcoma and breast (MCF-7), stomach (SGC-7901), and lung (A549) carcinoma cells [26]. Genistein has shown potential to reduce different types of cancer, such as breast, prostate, and ovarian [64]. This flavonoid induces breast cancer cell cycle arrest at the G2/M phase, followed by ROS-dependent apoptosis [64]. Other isoflavones, like daidzein, also induce apoptosis in breast cancer MCF-7 cells due to ROS production [65]. Naringenin can reduce ROS production and increase the activity of superoxide dismutase, catalase, and glutathione in cancer cells [66]. This flavanone has the potential to suppress metastasis and proliferation of MG-63 osteosarcoma cells [67]. Hesperidin can reduce the cell cycle progression in osteosarcoma MG-63 cells and induce apoptosis in several cancer cells like breast, ovary, prostate, and colon cancer cells [67]. Moreover, hesperidin displays antitumor and hepatoprotective effects against the development of hepatocellular carcinoma [67].

Epigallocatechin-3-gallate, a major flavonoid constituent in green tea, seems to be involved in cell growth arrest and death in prostate cancer [26]. Quercetin induces cell cycle arrest and growth inhibition on several malignant tumor cell lines in vitro, such as leukemia, colon, breast, and ovarian cancer cells [11]. Apigenin and luteolin change ROS signaling and induce apoptosis in several ovarian cancer cell lines (A2780, OVCAR-3, and SKOV-3) [68]. Cyanidin inhibits the proliferation and induces apoptosis of human epithelial colorectal adenocarcinoma cells (Caco-2) [69].

#### 4.2.3. Cardiovascular Protection

Flavonoids can act as cardio-protective agents by controlling oxidative stress (preventing the oxidation of low-density lipoproteins) and inflammation and by inducing vasodilation and regulating the apoptotic processes in the endothelium [62]. Flavonoids can interact with lipid metabolism and reduce platelet aggregation, preventing several cardiovascular diseases [70]. Some studies have demonstrated that quercetin, naringenin, and hesperetin have vasodilator properties, and naringenin reduces blood pressure and relaxation of vascular smooth muscles [6,62]. Isoflavones seem to protect against inflammatory vascular diseases, and quercetin has cardio-protective properties against heart injury and atheroprotective action associated with reducing oxidative stress [8]. Baicalin has been reported to improve cardiac dysfunction and reduce apoptosis in heart tissue [71]. Chrysin induces inhibition of platelet function, and genistein has antihypertensive properties [62]. Anthocyanins mitigate the risk of myocardial infarction in humans, improve systolic blood pressure, and decrease the levels of triglycerides as well as total and LDL cholesterol [72]. In addition, quercetin reduces systolic blood pressure and LDL blood concentration [7]. Several studies with acacetin, a flavone, demonstrated positive effects in the control of human arrhythmia [6].

#### 4.2.4. Antibacterial Action

Flavonoids can exert several mechanisms of action against bacteria. They can interfere with lipid bilayers by inducing bacterial membrane disruption and inhibit several processes such as biofilm formation, cell envelope synthesis, nucleic acid synthesis, electron transport chain, and ATP synthesis [73]. For instance, catechin, epicatechin and epigallocatechin gallate, and the flavonol quercetin seem to induce an oxidative burst, increasing ROS production that increases membrane permeability and damage [74]. Apigenin can destabilize the structure of the membranes by disordering and disorientating the membrane lipids, leading to membrane leakage [73]. The flavonoids, apigenin, chrysin, naringenin, kaempferol, quercetin, daidzein, and genistein interfere with biofilm formation, while the quercetin, luteolin, myricetin, and baicalein inhibit bacterial DNA replication [6,73]. Bacteria ATP synthesis can be inhibited by epigallocatechin gallate and baicalein [75].

#### 4.2.5. Antifungal Action

There are several antifungal mechanisms exerted by flavonoids, such as disruption of the plasma membrane, induction of several mitochondrial dysfunctions, and inhibition of cell wall formation, cell division, and RNA and protein synthesis [76]. Apigenin and baicalein can act as antifungals by controlling ROS species, reducing lipid peroxidation, and avoiding membrane disruption [6,77]. Some isoflavones, such as glabridin, can inhibit the synthesis of the main components of fungi cell walls, β-glucans, and chitin [78]. Quercetin can modulate several mitochondrial functions, like inhibition of oxidative phosphorylation and modifying of ROS production [79]. Apigenin interferes with the cell cycle, while myricetin, kaempferol, quercetin, luteolin, naringenin, and genistein inhibit DNA, RNA, and protein synthesis [80].

#### 4.2.6. Antiviral Action

Flavonoids can block the binding and penetration of viruses into cells, interfere with viral replication or translation, and prevent the release of the virus [81]. For instance, apigenin was demonstrated to be active against several DNA and RNA viruses, herpes simplex virus types 1 and 2, hepatitis C and B viruses, and the African swine fever virus by suppressing the viral protein synthesis [82]. Baicalein can impair avian influenza H5N1 virus replication in humans [83] and luteolin can have an antiviral effect on the reactivation of HIV-1 [83]. Epigallocatechin gallate exerts an antiviral effect throughout several steps of the HIV-1 life cycle [83]. Genistein can inhibit HIV infection of CD4 T cells and macrophages by interfering with HIV-mediated actin dynamics [84]. Kaempferol can also inhibit HIV replication in target cells [85] and block herpes simplex virus types 1 and 2 by attaching and entering the host cell [84]. The antiviral activity of quercetin, kaempferol, and epigallocatechin gallate against several influenza virus strains was demonstrated by Wu et al. [86].

## 5. Conclusions

In summary, flavonoids are important secondary compounds produced by plants with several functions related to growth and development and stress protection. The awareness of the beneficial properties of flavonoids for human health has triggered the increased consumption and interest in flavonoids’ uses in food processes and for therapeutic uses. Vegetables, flowers, and seeds are rich in flavonoids, and methodologies to extract these compounds from these natural sources have been developed to be used for other purposes, such as food additives and preservatives. The recognition of natural flavonoids as a good, safer source of antioxidants opens new perspectives to explore more of these compounds, focusing on new structures using new methodologies and technologies and exploiting other new natural sources.
